# A Consideration of Some of the Recent Work in Abdominal Surgery

**Published:** 1888-12

**Authors:** Joseph Price

**Affiliations:** Philadelphia, Pa.; Physician-in-charge of the Preston Retreat; 500 North Twentieth Street


Buffalo Medical and Surgical Journal

Vol. XXVIII. DECEMBER, 1888. No. 5

### Original Communications.

### *A CONSIDERATION OF SOME OF THE RECENT WORK IN ABDOMINAL SURGERY.*

By JOSEPH PRICE, M. D., Philadelphia, Pa.
Physician-in-charge of the Preston Retreat.

Every movement of scientific progress has its period of experimentation, its period of probation, during which it is weighed and its value determined; and, finally, the time of its adoption as a scientific procedure, or of its abandonment as worthless or inadvisable.

All of these stages have been passed through by abdominal surgery. Its recognition is now undisputed, though some few of the various operations proposed within the domain are still regarded as questionable in certain cases and under certain conditions. Under this latter head may be mentioned hysterectomy for uterine cancer, and splenectomy.

The operations of the various pathological conditions of the uterine appendages form, by all odds, the greater portion of abdominal surgery. The variety of conditions met here are almost past enumeration, each case varying in a manner peculiarly its own, both as to its exact causation and in its relation to other abdominal viscera. Pus-tubes may be one-sided or bilateral, and the same is true of ovarian cysts. These may be suppurating or simple, or gangrenous by reason of a twisted pedicle. Their adhesions may be nothing, varying from this to universal. As to the treatment of pus-tubes, now that their existence is acknowledged by all save a doubting few, who, unable to recognize them, therefore discredit their existence. Removal at once on discovery is the fast and firm principle. This is established past question in the minds of a majority of operators. The same may be said of ovarian tumors.

Cysts of the broad ligament are also complicated or simple. Tubal disease may be found present with both ovarian and ligamental tumors. Hydrosalpinx and hematosalpinx, while we are often not able to differentiate before operation, may also complicate ovarian disease. Dermoid cysts also afford similar complications to those of other cysts, and are quite prone to suppuration. Tubal pregnancy is of late occupying a prominent place in operative procedures, as affording the greatest scope for surgical ingenuity, while it, at the same time, is not encouraging unless taken early and treated promptly.

Its diagnosis, so much discussed, is now, by common consent, regarded as doubtful before rupture, and if made as accidental, a happy-go-lucky guess, which is harmless, and satisfactory to the operator. Mr. Tait's remarkable experience in these cases is worth that of all other operators combined, and his opinion, to my mind, is of like value and worthy of the greatest respect. An expression of his opinion in regard to the diagnosis of these cases may not be without interest. He says: "The strangest thing of all to me is, that in the enormous experience I have now had of tubal pregnancy, I have never but once been called upon even to make an examination until the rupture had occurred, and in that case there was neither history nor symptoms which enabled me to do more than determine there was tubal occlusion. Not, indeed, until the rupture occurred and the abdomen was opened was a diagnosis possible. Under these circumstances, I think I may be excused for maintaining a somewhat skeptical attitude concerning the correctness of the diagnosis of those gentlemen who speak so confidently of making certain diagnoses of tubal pregnancy before the period of rupture, and who speak with equal confidence of curing the cases by puncture, either simple, medicated, or electrolytic. I wish to say that, after the period of rupture, a diagnosis can and has been made, in my own experience, in a majority of these cases. The great bulk of these utterances may stand very well in society discussions, or in library papers, but they will not stand the test of bedside experience."

Operations for the removal of gall-stones offer great inducement for successful treatment. Treatment of ileo-cecal abscess or appendicitis by the abdominal section offers a direct method of dealing with this hitherto usually fatal or chronic affliction. When the lesions are clear, the lateral incision is the choice. The median section is, for many reasons, often advisable, and, when there is any doubt as to the exact condition of the case, is, perhaps, the best. The closure of the incision should be insisted upon, and drainage carefully established. To insist on strict antisepsis in an operation, and then to leave the abdomen open, appears a contradiction in terms, and illogical.

A method of treatment of pelvic abscess, not in accord with the generally received methods, is that reported by Professor Martin in the May number of the *American Journal of Obstetrics.* It is to treat the abscess by puncture through the vagina, and where there is difficulty or uncertainty in fixing and locating the tumor, to open the abdomen, disengage the mass from its adhesions, bring it down within reach of the trocar, and, finally, puncture and introduce a drainage-tube. The Professor reports the three cases so operated upon, and says: "The wound is not washed out, and the tube remains for months after the patient has gotten out of bed."

A brief discussion of this method seems not out of place. Any operator who, fearing to open the peritoneum, would prefer to puncture through the vagina, would have some measure of reason on his side. But to open the abdomen to free a mass from its adhesions, in order to bring it within reach of a trocar through the vagina, seems too fantastic in its conception to be entertained for a moment.

As to Professor Martin's method of locating and fixing the tumor by abdominal section, making vaginal drainage, and closing the abdomen without attempt at removal of the tumor, I cannot but disapprove of it.

In this case, only the operator's name makes it possible for such a suggestion to receive a following. When a man of Prof. Martin's acknowledged ability, operative dexterity, and skill, makes a suggestion, and gives it his sanction, it is taken as the gold of his experience, with the stamp of his approval.

Ordinarily, this is worth much. But even genius is liable to err; and I believe that before long Professor Martin himself will relegate this procedure to oblivion, along with the other abandoned operations of our profession, and, if suggesting nothing new to replace it, go back to the older and, I am convinced, the better plan of removal and drainage through the original abdominal incision.

If I open an abscess through any wall, why not drain it through the original incision? To open the abdomen, simply to bring a mass within reach of a trocar after it has been freed from its adhesions, is on a par with making an incision over a diseased bone; carefully freeing the sequestrum, taking care also not to remove it; diligently suturing the incision; making a second incision, by whatever means fancy may dictate; introducing a drainage-tube, and allowing the dead and stinking mass slowly to come away.

I am sure one method is just as logical as the other.

The idea, too, of allowing a woman to carry a drainage-tube for months, when a section, with the removal of the mass, will allow her in the majority of instances to go about well, free from such annoyance and discomfort, in three weeks, is preposterous. We are too far from Egypt and the pyramids to plough our ground with sharpened sticks.

Whatever improvement is to be added to the technique of any operation, should be in the line of progress, and nothing should be proposed for the sake of novelty and innovation. Originators are few, imitators are many, and the harm done to suffering humanity by those who follow without thinking and without special training, simply taking the dogma of a leader, is incalculable.

The treatment of any pelvic abscess simply by puncture and drainage through the vagina, is at best a slow procedure, and, I

fear, will give not a measure of success comparable with the discomfort it so often entails.

A discussion of the recent work in laparatomy would, however, be incomplete without some allusion to a peculiar fact concerning the operators in this branch of surgery. I refer to the comparative youth of many of our abdominal operators. As a man, neither very young nor yet in the sere and yellow leaf, I may refer, perhaps, more impartially to these men, leaving their disparagement—both by their elders, on account of their youth, and by some of their co-workers of equal age, on account of a feeling, so often the deep damnation of our profession, an overpowering jealousy—behind me, resurrecting it, only to bury it, deeper I trust, in the ditch, that the merit of these men have digged for it.

I hear cried on all sides, "There are too many men at work." To this I can say "Amen," but not in the sense of the complainants. The work of many men now beginning is the uncompleted and imperfect work of those who have preceded them. There are too many imperfect workers with experience to give them prominence; there are too few conscientious workers, young or old, to recognize and relieve the suffering women in the courts and alleys of this and other cities and towns. The old worker who will not learn is more dangerous, and his work more to be deplored, than that of the young man, giving time for careful training, watching and working, that he may fail in nothing to perfect his art.

Once teach the younger operators, or the young men about to devote themselves to this sort of work, that this early recognition of abdominal disease is the keynote of success in all its branches, and we will have no crying need, as it is our shame still to have, of a Bantock and Tait expostulating against the delays of prominent operators, as dangerous and often fatal.

Mr. Tait's second series of 1,000 cases just published, proves by indisputable statement of facts, together with incontrovertible logic, that early operation, and completed operations, give patients the best possible chance of recovery.

When an operator has a success indicated by a mortality of only 5.3 per cent. in a thousand cases, his dictum must be respected even by his enemies.

A full discussion of all progress would be impossible in the limits of this paper, and it is not intended to be exhaustive. At a future time I trust to consider a few of the more important abdominal operations at greater length.

In the light of the originality of its conception and importance, it would be unjust to conclude this paper without referring to the method of using hydrogen gas in the localization of intestinal wounds. This idea offers a still further field for investigation, and renders the surgery of gun-shot wounds at once simpler and safer.

500 North Twentieth Street.

